# Assessing quality of newborn care at district facilities in Malawi

**DOI:** 10.1186/s12913-020-5065-2

**Published:** 2020-03-18

**Authors:** Kondwani Kawaza, Mai-Lei Woo Kinshella, Tamanda Hiwa, Jenala Njirammadzi, Mwai Banda, Marianne Vidler, Laura Newberry, Alinane Linda Nyondo-Mipando, Queen Dube, Elizabeth Molyneux, David M. Goldfarb

**Affiliations:** 1 Department of Pediatrics and Child Health, College of Medicine, University of Malawi, Blantyre, Malawi; 2 College of Medicine, IMCHA Project, Blantyre, Malawi; 3 Department of Obstetrics and Gynaecology, BC Children’s and Women’s Hospital and University of British Columbia, Vancouver, Canada; 4 Department of Health Systems and Policy, School of Public Health and Family Medicine, College of Medicine, University of Malawi, Blantyre, Malawi; 5 Queen Elizabeth Central Hospital, Pediatrics, Blantyre, Malawi; 6 Department of Pathology and Laboratory Medicine, BC Children’s and Women’s Hospital and University of British Columbia, Vancouver, Canada

**Keywords:** District hospitals, Quality of care, Malawi, Neonatal care

## Abstract

**Background:**

Malawi is celebrated as one of the few countries in sub-Saharan Africa to meet the Millennium Development Goal of reducing under-5 mortality by two-thirds between 1990 and 2015. However, within this age range neonatal mortality rates are the slowest to decline, even though rates of facility births are increasing. Examining the quality of neonatal care at district-level facilities where most deliveries occur is warranted.

**Objective:**

The objective of this paper is to evaluate the quality of neonatal care in three district hospitals and one primary health centre in southern Malawi as well as to report the limitations and lessons learned on using the WHO integrated quality of care assessment tool.

**Methods:**

These facility assessments were part of the “Integrating a neonatal healthcare package for Malawi” project, a part of the Innovating for Maternal and Child Health in Africa (IMCHA) initiative. The WHO integrated quality of care assessment tool was used to assess quality of care and availability and quantity of supplies and resources. The modules on infrastructure, neonatal care and labour and delivery were included. Facility assessments were administered in November 2017 and aspects of care were scored on a Likert scale from one to five (a score of 5 indicating compliance with WHO standards of care; one as lowest indicating inadequate care).

**Results:**

The continuum of labour, delivery and neonatal care were assessed to identify areas that required improvements to meet standards of care. Critical areas for improvements included infection control (mean score 2.9), equipment, supplies and setup for newborn care in the labor ward (2.3), in the surgical theater (3.3), and nursery (3.4 nursery facilities, 3.0 supplies and equipment), as well as for management of sick newborns (3.2), monitoring and follow-up (3.6). Only one of the 12 domains, laboratory, met the standards of care with only minor improvements needed (4.0).

**Conclusion:**

The WHO integrated quality of care assessment tool is a validated tool that can shed light on the complex quality of care challenges faced by district-level health facilities. The results reveal that the quality of care needs improvement, particularly for sick and vulnerable newborns.

## Background

### Maternal and child health trends in Malawi

Malawi is celebrated as one of the few countries in sub-Saharan Africa to meet the Millennium Development Goal of reducing under-5 mortality by two-thirds between 1990 and 2015 [1]. Under-5 mortality declined from 234 deaths per 1000 live births in 1992 to 63 deaths per 1000 live births in 2015–16, representing a 73% decrease over a 24-year period [2]. This reduction in mortality was attributed to early adoption of high impact interventions in communities and basic obstetric and neonatal care to address major causes of child death, such as treating pneumonia, diarrhoea and malaria, promotion of vaccines and insecticide-treated bed-nets, supplementary nutrition programme, facility births and prevention and treatment of HIV [1]. However, while child mortality has decreased for all age groups, neonatal mortality rates are declining the slowest, even though rates of facility births are increasing [2]. Neonatal mortality declined from 41 in 1992 to 27 deaths per 1000 births in 2004 [2]. Over a decade later, the 2016 neonatal mortality rate in Malawi remains at 27 deaths per 1000 live births [2]. Preterm birth is a risk factor in over 50% of all neonatal deaths [3], and at 18%, Malawi has the highest recorded rate of babies born prematurely in the world [4]. Preterm infants are vulnerable to feeding difficulties because of immature sucking and swallowing reflexes, have a higher likelihood of developing breathing problems and have body temperature instability [5, 6]. In Malawi, children reported to be small or very small are twice as likely to die in the first month of life as children reported to be average or larger (44 versus 22 deaths per 1000 live births) [2].

### Importance of quality of care at facilities

Public health initiatives and improved basic obstetric and neonatal care, including breastfeeding, warmth, hygiene, antibiotics, and resuscitation, can lead to major reductions in neonatal deaths [6]. However, preterm births may require targeted and intensive care. While there are some community-level public health approaches for the care of premature babies, the highest impact interventions require facility-based services [6, 7]. Neonatal intensive care includes incubators, ventilation, and overall increasing complexity of care [6, 7]. The rapid increases in health facility births in many countries in sub-Saharan Africa and South Asia, including Malawi where 55% of births were delivered in health facilities in 2000 compared to 91% in 2016 [2, 8], has not been accompanied by similar increases in quality of care [9]. In Malawi, complications of preterm birth, severe infection and birth asphyxia account for 89% of all neonatal mortality, which may be reduced by quality facility-based care. However, the quality of newborn health care services in Malawi was found to be lower than for other health services at health facilities [10]. The high rates of survival of premature babies in high-income countries demonstrates that it is possible to effectively reduce morbidity and mortality among this vulnerable population, but there remains a gap in implementing effective interventions in low- and middle-income countries (LMICs), particularly at the district-level where the burden, but also the potential for impact, is highest [7]. In general, improving newborn survival is possible with simple, immediate, facility-based interventions, including the provision of warmth with immediate drying, stimulation and skin-to-skin contact for the newborn, resuscitation with bag and mask, early initiation of exclusive breastfeeding, hygienic cord care and management of respiratory complications [11]. Consequently, it is important to understand quality of care for newborn health at facilities in Malawi, particularly at the rural district-level.

### District-level hospitals

While it is important to strengthen all levels of hospital care, the district hospital has been highlighted as a neglected component of health systems in LMICs [12]. District hospitals are central hubs for higher-level clinical care in rural communities and a key location for medical referrals and health worker training [12]. It is especially important to improve quality of care for neonatal health at district-level hospitals in Malawi because most deliveries are happening at the district hospitals, not tertiary facilities. The objective of this paper is to evaluate the quality of neonatal care in three district hospitals and one primary health centre in southern Malawi as well as to report the limitations and lessons learned on using the WHO Integrated Maternal, Neonatal and Child Quality of Care Assessment and Improvement Tool (version April 2014).

## Methodology

### Integrating a neonatal healthcare package for Malawi

These facility assessments were carried out within the “Integrating a neonatal healthcare package for Malawi” project, which is part of the Innovating for Maternal and Child Health in Africa (IMCHA) initiative, funded by the Canadian International Development Research Centre (IDRC), Global Affairs Canada (GAC) and the Canadian Institutes for Health Research (CIHR). With a focus on implementation science and quality improvement, the project seeks to strengthen neonatal care at health facilities in low-resource settings, through understanding the roll out of low-cost technologies and locally appropriate innovations. The facility assessments serve as a baseline for the current routine care environment and capacities for neonatal care at district-level facilities in Malawi.

### Adaptation of the WHO integrated quality of care assessment tool

In 2014, the WHO Integrated Maternal, Neonatal and Child Quality of Care Assessment and Improvement Tool was developed from two existing survey instruments, the Health Facility Survey tool to evaluate the quality of care delivered to sick children and the Hospital Care for Mothers and Newborn Babies quality assessment and improvement tool [13]. The Health Facility Survey, developed by the WHO Department of Child and Adolescent Health and Development in 2003, is based on the Integrated Management of Childhood Illness (IMCI) clinical guidelines [14] and has been used in various settings including rural Ghana [15] and Afghanistan [16]. The Hospital Care for Mothers and Newborn Babies quality assessment and improvement tool is a comprehensive systematic, standards-based, participatory approach instrument developed by the WHO Regional Office for Europe first published in 2009, revised in 2014 [17] and has been employed both in Europe [18] as well as in lower resource settings [19]. Integration of the two surveys generated a generic assessment tool to evaluate quality of care for mothers, babies as well as children in hospital settings. The tool was refined based on standards derived from the WHO Pocketbook of Hospital Care for Children and the WHO Integrated Management of Pregnancy and Childbirth and prioritization of key areas with the highest impact on improving quality care [20, 21]. Key priority areas included triage, hygiene, emergency and first-line drug availability, availability of treatment guidelines and management of emergency, common and routine conditions [20, 21]. The comprehensive facility assessment was designed to be both a management and evaluation tool and in contrast to other facility assessments, it examined quality of care as well as quantity and availability [13]. The 2014 WHO Integrated Maternal, Neonatal and Child Quality of Care Assessment and Improvement Tool [20] was shared with us by Malawian Ministry of Health partners and it was used for the first time in Malawi in 2015 in 35 health facilities in five districts [13]. To the best of our knowledge, this is the second time it has been used in Malawi. It has also been used by UNFPA in Sierra Leone [22–25] and adopted as the assessment tool by the WHO Regional Office of South-East Asia [21].

The complete tool has four modules related to A) infrastructure, B) maternal, C) newborn and D) paediatric care. Module A gathers basic hospital statistics and information about infrastructure, ward layout and the organization of care including staffing. Modules B, C and D assess quality of care and included sections on emergency care, inpatient care, infection control and supportive care, essential drugs, equipment and supplies, case management, monitoring and follow-up. While Smith and colleagues [13] evaluated using the full tool, this study focuses only on neonatal care. Consequently, only the modules on infrastructure, neonatal care and maternal care, as related to labour and delivery, were included. There were twelve key areas of care assessed covering infrastructure (A), laboratory (A), labour and delivery facilities (B), caesarean section facilities (B), prevention and management of preterm labour (B), nursery facilities (C), infection control (C), supportive care of sick neonates (C), neonatal care equipment and supplies (C), routine neonatal care (C), case management of the sick newborn (C) and monitoring and follow-up of sick newborns (C) (see Additional file 1). This reduced the length of the assessments by about two thirds.

### Study sites

This study was conducted in the Southern region of Malawi. Three districts were chosen in consultation with the Malawi Ministry of Health because they represent a variety of health management structures available in Malawi. District 1 and 3 each have a government district hospital while District 2 features a Mission Hospital that operates as the region’s district hospital. Mission hospitals are under the umbrella of the Christian Health Association of Malawi (CHAM) and they provide between 30 and 40% of the health care in the country [26]. Essential health care, which includes maternal and child health services, are free to patients at Mission Hospitals under a service agreement with the government. Because the government does not have a district hospital in District 2 and relies on the Mission Hospital, an assessment was also conducted at the primary health centre in the main town, where the district management is located. Districts were also selected because they represent different geographic zones. District 1 and 3 are in the southwest and District 2 is in the southeast zone. District 2 is also more remote. The three hospitals represent a spectrum of district-level facilities for implementation.

### Administering the facility assessment

The four steps in conducting the hospital assessment outlined by the Integrated Maternal, Neonatal and Child Quality of Care Assessment and Improvement Tool include an introductory meeting with hospital administrators and staff, a walk-through to assess the hospital organization and areas to conduct the assessment, conducting the assessment and then a meeting between data collectors to debrief [20, 21]. The research team initially introduced the project and its objectives to the district health management and obtained permission. Five IMCHA employed research nurses were trained to do the assessment and then deployed in November 2017. This involved observations of practices and availability of infrastructure, equipment and supplies as well as interviewing relevant health professionals, such as the nurse-in-charge and laboratory technicians.

Aspects of care observed by data collectors were scored on a Likert scale from one to five. A score of five indicated good practice complying with recommended standards of care, a score of four indicated minor need for improvement, a score of three indicated some need for improvement, a score of two indicated considerable need and a score of one indicated totally inadequate care or a potentially life-threatening practice [13, 20, 21]. Open-ended comments were elicited from data collectors following each section to reflect on the strengths and weaknesses as observed. The assessments took between 3 days to 2 weeks to complete, depending on the availability of hospital staff for observations and interviews. Approval was obtained from the research ethics boards of the Malawi College of Medicine (P.08/15/1783) and the University of British Columbia (H15–01463-A003).

### Data analysis

Results were recorded on paper, scanned and transferred to a REDCap database. Each facility assessment was manually entered by two independent people and results compared to reduce inaccuracies in data entry and interpretation. Embedded in the tool, summary scores for each area of care were calculated as an average of responses in the section. Basic descriptive statistics completed on Excel were used to analyze summary scores for each area of care. Following Smith and colleagues [13], standards were considered to have been met if scored four or five.

## Results

### Facility characteristics

Two of the three hospitals, District 2 and 3, had a separate ward for admitting newborns with 10–12 beds each. The hospital in District 1 admitted neonates to the labour and postnatal wards. However, nurseries at District 1 and 3 hospitals were under renovation at the time of assessment. The primary health centre did not admit newborns and newborns requiring specialized care - including supportive care, case management, monitoring and follow-up of sick newborns, and neonates requiring specialized care - were referred to the Mission Hospital. The primary health facility did not have a separate nursery and facility data were collected in the general postnatal ward where babies were observed with their mothers for a few hours before discharge. Caesarean deliveries were separate from the labour ward in all three district hospitals; the primary health centre did not conduct caesarean deliveries.

None of the facilities had a full time obstetrician-gynaecologist. One hospital (District 1) had a clinical officer trained in obstetrics and gynaecology while the other three sites (District 2 Mission Hospital and primary health centre, District 3 hospital) had visits by an obstetrician approximately once a month. However, staff reported that the obstetrician had not been visiting of late in District 3. Caesarean deliveries were done by clinical officers and general medical officers, in some cases, who had been trained ‘on the job.’ None of the facilities had a full-time paediatrician. The primary health centre had a visiting paediatrician once a month while the three district hospitals did not have a visiting paediatrician. District-level facilities were staffed by clinical officers, nurses/midwives and lay health workers (Table 1).

**Table 1:**
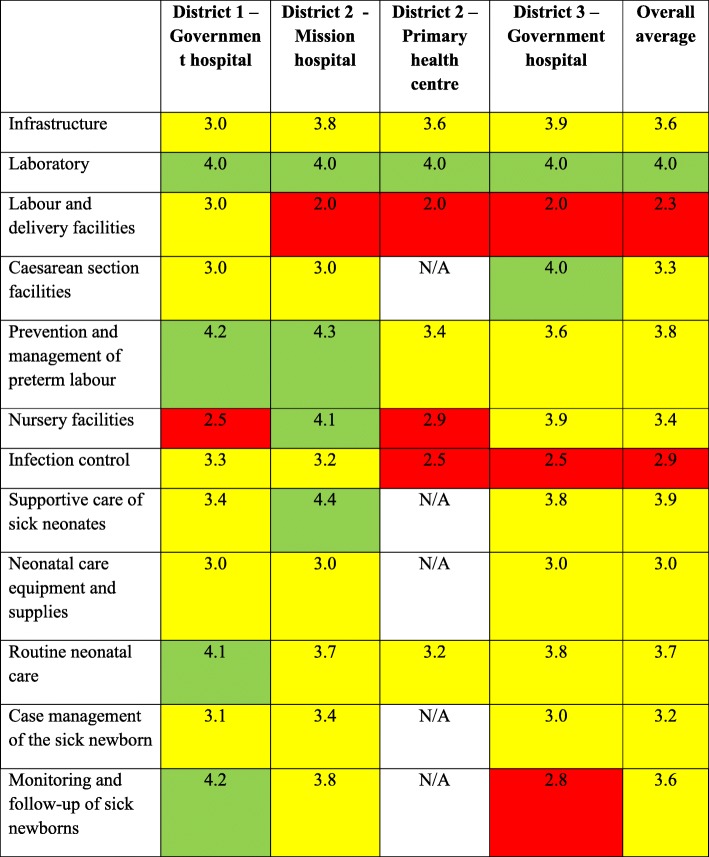
Quality of care scores

*5 being good practice complying with standards of care; 4 showing little need for improvement to reach standard of care; 3 meaning some need for improvement to reach standards of care; 2 indicating considerable need for improvement to reach standards of care; and 1 being services not provided, totally inadequate care or potentially life-threatening practices* [20]

### Infrastructure

The average score for the four facilities was 3.6, indicating that all facilities had some need for improvement to meet standards of care. The site scores ranged from 3.0 to 3.9. Electricity was not continuously available in any site and back-up power systems were insufficient. For example, a solar power system in one site was not sufficient to operate heavy equipment and an additional diesel generator was needed for the operating theatre and nursery. Another site noted that water was usually not available when there was a power cut. A third site noted that while a back-up power system was available, it was not in use most of the time. While there was often a functioning fridge available for drugs or vaccines, it may be located in a different department. There was a lack of soap and disinfectants in three out of the four facilities.

### Laboratory

All of the laboratory facilities scored 4.0, placing them in the category of little improvement needed to meet standards of care. Most tests were available in the laboratory, including blood glucose, haemoglobin, HIV, syphilis, urine dipstick, urine microscopy and full blood count testing. There were some gaps for hematocrit (PCV) and bilirubin testing across sites and testing for blood grouping and Rhesus antibody was only available at the hospital facilities. However, some key tests for management of sick newborns were missing - none of the four sites had blood gas analysis or blood cultures available. All available tests were said to be reported in under an hour. Space was frequently limited in the laboratory and one site noted that there was no back-up power supply for the laboratory.

### Labour and delivery, caesareans and nursery facilities

For labour and delivery facilities, the average score was 2.3, indicating considerable need for improvement to meet standards of care. The hospital in District 1 had a score of three while the other three sites scored two. Inadequate lighting, limited space and lack of sterile gloves, a heating source for neonates and equipment for neonatal resuscitation were areas of concern in the labour and delivery ward. In two of the three hospitals, the oxygen concentrator was shared by the whole maternity department or borrowed from the nursery. The average score for caesarean section facilities for the three hospitals was slightly better at 3.3, indicating some need for improvement to meet standards of care. District 3, where the most deliveries were recorded, had a score of 4.0. Most equipment and supplies were available and at one site the theatre was well arranged. Heating lamps for newborns and an infusion pump were not available in all sites.

For nursery facilities, the average score was 3.4, indicating some need for improvement. There were considerable differences between the sites ranging from 2.5 to 4.1, with the Mission Hospital scoring the highest. Lack of running water was a problem in two of the three hospitals, leading to unclean toilets. Understaffing was explicitly highlighted in the comments in one hospital. Two of the three hospitals did not have mosquito nets in the nursery despite being a malaria endemic area. The Mission Hospital was clean but only the staff had access to handwashing stations.

### Prevention and management of preterm labour

The overall average score for prevention and management of preterm labour was 3.9. District 1 scored over four, indicating little improvement needed, while District 2 and 3 facilities scored 3.4–3.9, indicating the need for some improvement. When interviewed, staff were knowledgeable around managing preterm labour and the use of tocolytic drugs. Corticosteroids were given to the mother to improve foetal lung maturity and chances of neonatal survival, if less than 34 weeks gestation and medical staff were prepared to care for and resuscitate a preterm or low birth weight baby if necessary. However, protocols and guidelines on the management of preterm labour were largely not available, vacuum extraction was not avoided and most of the preterm labour was not being prevented. Additionally, records on preventing labour or antenatal administration of corticosteroid were not kept.

### Infection control

None of the district-level facilities met standards of care for infection control and the average score was 2.9, indicating considerable need for improvement. The scores ranged between 2.5–3.3. At the three public health facilities, hand hygiene was followed infrequently and soap/disinfectants were not available. Though the private mission hospital had a well-organized handwashing station and guidelines posted, hand hygiene was not practiced regularly. Gloves were sometimes used instead of hand hygiene. Sterile gloves were not available at the primary health centre. While infection control policies were sometimes available, they were rarely put into practice. Routine disinfection of the premise were scheduled but irregularly preformed due to staff shortages. Additionally, one site noted that disinfection was compromised by facilities remaining open. In the three hospitals, a routine policy of changing dress and footwear by staff in the operating room was not observed.

### Essential drugs, equipment and supplies for neonatal care

The three hospitals each scored 3.0, indicating some need for improvement. Incubators, heated mattress cots, multi-function monitors and appropriately sized nasogastric tubes for preterm babies were not available. A radiant warmer and digital scale were available at the hospital in District 3 but located in the labour ward. One phototherapy lamp was available, one to two functioning oxygen concentrators, appropriate sized face masks, and one to two functioning CPAP machines, pulse oximeter and suction apparatus were available at each hospital. Glucometers were available but at one site there were no glucostix to perform the test. Some oxygen concentrators and CPAP machines were not functioning and not all staff were trained to use the CPAP machine. Appropriate-sized self-inflating bags were available though of insufficient number and not always functional. Thermometers were reported to be available but mostly staff kept their own personal thermometers.

Of the drugs, penicillin, ceftriaxone and gentamicin were the most available antimicrobials, and phenobrbitone the available anticonvulsant. IV glucose and ferrous sulphate were often available. Drugs that were available were not close to expiry but there was often minimal stock. Vancomycin, surfactant, sodium bicarbonate, chlorohexidine for cord care, vitamin D, vitamin K and IV calcium were not stocked at the district hospitals. Most of the drugs are kept at the pharmacy rather than in the nursery.

### Routine neonatal care

The overall average score for routine neonatal care was 3.7, indicating some need for improvement to meet standards of care. The hospital in District 1 scored 4.1 while the other facilities scored between 3.2–3.8. Early and exclusive breastfeeding (4.6), neonatal resuscitation (4.0), screening, prevention and management of vertically transmitted infectious diseases (4.0), and counselling for mothers (4.0) met standards of care with minor improvements needed. However, newborn assessments were not complete and newborns’ breathing and body temperatures were irregularly monitored. Additionally, there were failures to document breastfeeding, jaundice and the mothers’ health.

### Supportive care, case management, monitoring and follow-up of sick neonates

The three hospitals had an average score of 3.9 in supportive care for sick neonates, indicating the need for some improvements. Only the private mission hospital met the standards of care with little improvements needed (4.4). The provision of IV fluids and blood transfusions were rare and none were observed during the assessments. However, staff reported that IV fluids and blood transfusions were used when indicated. Drugs were also given with a clear indication and routine use of sedatives was not the norm. Blood glucose was poorly monitored.

The three hospitals had an average score of 3.1, ranging between 3.0–3.4, for case management of sick neonates, indicating the need for some improvements. In particular, there was poor recognition and treatment of jaundice and management of convulsions. There were also problems in diagnosing neonatal sepsis because of the lack of blood and urine cultures. Guidelines for management of convulsions and jaundice were not available and feeding sick neonates were not recorded or monitored routinely. The wards practiced kangaroo mother care and there was good maintenance of room temperature at 25-28 °C.

The three hospitals had an average score of 3.6 in monitoring and follow-up of sick newborns, indicating that some improvements are needed. However, there was variation across the three sites. The hospitals in District 3 scored 2.8, District 2 scored 3.8 and District 1 scored 4.2 indicating substantial, some and little need for improvement, respectively. Monitoring by nurses met the standards with little improvement required (4.0) but reassessment by physicians required substantial improvements to meet standards of care (average score = 2.9). Daily reassessments by a doctor were not completed, with the exception of the Mission Hospital in District 2, though a doctor did not review sick neonates or new admissions on weekends and holidays (Table 2).

**Table 2:** Key quality of care gaps highlighted in the facility assessment

Infrastructure	• Lack of reliable electricity • Lack of soap and disinfectants
Laboratory	• Blood gas analysis and blood cultures not available • Limited space
Labour and delivery facilities	• Inadequate lighting • Limited space • Lack of sterile gloves • Lack of a heating source for neonates • Lack of equipment for neonatal resuscitation
Caesarean section facilities	• Lack of a heating source for neonates • Lack of equipment for neonatal resuscitation
Prevention and management of preterm labour	• Lack of protocols and guidelines on the management of preterm labour • Lack of records on preventing labour or antenatal administration of corticosteroid
Nursery facilities	• Lack of running water, unclean toilets, patient access to handwashing stations • Understaffing
Infection control	• Poor hand hygiene practice, lack of soap and disinfectants • Gloves sometimes used instead of hand hygiene • Infection control policies and routine disinfection of the premise were rarely practiced
Supportive care of sick neonates	• Poor monitoring of neonates’ blood glucose
Neonatal care equipment and supplies	• Lack of incubators, heated mattress cots, multi-function monitors and appropriately sized nasogastric tubes for preterm babies • Some oxygen concentrators and CPAP machines were not functioning and gaps in training on their use • Insufficient number of appropriate-sized self-inflating bags for resuscitation • Vancomycin, surfactant, sodium bicarbonate, chlorohexidine for cord care, vitamin D and IV calcium not available
Routine neonatal care	• Newborn assessments not completed • Irregular monitoring of newborns’ breathing and temperatures • Poor documentation
Case management of the sick newborn	• Lack of guidelines for management of convulsions and jaundice • Lack of blood and urine cultures for neonatal sepsis diagnosis • Feeding sick neonates were not recorded or monitored routinely
Monitoring and follow-up of sick newborns	• Poor reassessment by physicians

## Discussion

In the evaluation of neonatal quality of care at four district-level health facilities in southern Malawi using the Integrated Maternal, Neonatal and Child Quality of Care Assessment and Improvement Tool, only one of the 12 domains, laboratory, met the standards of care with minor improvements needed. There were critical gaps in labour and delivery facilities as well as infection control, which both required substantial improvements to meet standards of care. Caesarean section facilities, nursery facilities, neonatal care equipment and supplies and case management of the sick newborn were also critical areas for improvement. Infrastructure, prevention and management of preterm labour, supportive care of sick neonates, routine neonatal care and monitoring and follow-up of sick newborns required some improvement to meet standards of care but were getting closer to the score of four out of five.

Smith and colleagues [13] implemented the full tool in five districts in Malawi, including two districts from southern Malawi, and found similar key areas for improvement. This included, in the domains of neonatal care equipment and supplies (3.0 our study vs 2.9 Smith et al), infection control in neonatal care areas (2.9 our study vs 3.3 Smith et al), case management of the sick newborn (3.2 both studies) and nursery facilities (3.4 our study vs 3.2 Smith et al). Prevention and management of preterm labour was higher in the facilities surveyed than those reported by Smith et al. (3.9 vs 3.0) though this may be because Smith et al. [13] surveyed a wider range of facilities. Monitoring and follow-up of sick neonates, especially with physician reassessment, and infrastructure, particularly of electricity and running water supplies, were also highlighted as key challenges that were previously not discussed by Smith et al. [13]. At the time of the assessments, there were daily power outages of six to 8 h per day, which compromised ability to use necessary medical equipment, have running water and carry out lab tests. Though back-up power supplies were available, these were not sufficient to operate the entire hospital and often prioritised for the theatre. Often there were delays in obtaining permission to start the generator.

### Complexities in key areas of newborn care

Neonatal jaundice is a key concern for premature babies as the immature liver cannot metabolise bilirubin efficiently, and high levels carry the risk of irreversible brain damage [27]. A closer look at the questions around neonatal jaundice and management with phototherapy demonstrates some complexities of care. Bilirubin testing was available in each of the districts and the average time to results were remarkably fast, about 45 min to 1 h. However, low scores on the questions pertaining to jaundice and phototherapy contradict the high scores in availability of lab testing. There were gaps in procedures to check bilirubin levels, examining babies for jaundice and problems with the guidelines and supply of phototherapy. Only one phototherapy lamp was available at each of the three district-level hospitals and the phototherapy lamps were irregularly checked for correct functioning. Consequently, the fast bilirubin testing may also be associated with low ordering of the tests from the wards. Additionally, the private district hospital could not test bilirubin levels but could provide phototherapy while the primary health centre had bilirubin testing available but did not manage neonatal jaundice.

A closer look at management of respiratory complications also sheds light on complexities of care. Currently CPAP is available in all district hospitals in Malawi, which itself is an accomplishment for scaling up important neonatal care technologies. The widespread availability of CPAP is unusual for secondary level facilities in sub-Saharan Africa. However, findings of the management of respiratory complications and the case management of sick newborns revealed a number of concerns. While CPAP systems were available and all facilities had high scores for managing respiratory distress, there was a lack of guidelines for the use of oxygen in preterm babies, oxygen needs were not routinely assessed using a pulse oximeter and at one site, two of three oxygen concentrators were not functional.

Kangaroo mother care (KMC) met standards of practice in each of the district hospitals; however, there were critical gaps in infant warming devices such as incubators, radiant warmers and heated mattresses/hot cots. Infant warming devices are recommended for unstable newborns weighing 2000 g or less at birth or stable newborns who cannot be given KMC [28].

A key issue highlighted in this assessment, also noted by others [13], is the lack of effective infection prevention and control. In many low resource settings this has been associated with very high rates of nosocomial (hospital onset) infections, generally 3 to 20 times higher than those seen in babies born in hospitals in industrialized countries [29]. The rise of antimicrobial resistant organisms often resistant to first and second line antibiotics [30] coupled with the lack of the availability of basic microbiology testing could further amplify the impact of gaps in infection prevention and control.

### Quality of care and impact on neonatal mortality

A cross-sectional survey exploring obstetric facility quality and neonatal mortality in Malawi found that delivery facilities are accessible and used but documented poor quality standards, especially in rural areas [31]. Health facilities in the lowest quartile of quality were associated with 23 more neonatal deaths per 1000 live births in Malawi [31]. Facility assessments conducted in Malawi [13, 31, 32], including the one presented here, highlight the potential impact of quality care on neonatal mortality, which can be further understood through historical modelling of phases of neonatal mortality reduction. Historical modelling in the United Kingdom and United States during the twentieth century described the first phase of reducing neonatal mortality rate (NMR) from 40 to 30 in association with public health interventions, such as sanitation and hygiene and facility births [6, 33]. In Malawi, the NMR fell from 41 in 1992 to 27 in 2004 as hospital births began to outnumber home births in the early 2000s [2]. However, in the last 16 years, the rate of decline has stagnated around 27 deaths to 1000 live births, which may be associated to the critical gaps in case management of sick newborns and infection control. The second phase of NMR reduction from 30 to 20 has been associated with improved obstetric and neonatal case management, especially improvements in newborn thermal care with the introduction of incubators and promoting breastfeeding. Antibiotics and infection management furthered the decline from 20 to 15 NMR [33]. This facility assessment found that incubators, warming mattresses/cots and radiant warmers were not widely available and lactation support and infection control were not uniformly followed. To improve the NMR of Malawi from 27 per 1000 livebirths, significant gaps in infection control practices must be addressed.

Historical modelling found a third phase of NMR decline from 15 to 5 per 1000 live births associated with improved neonatal intensive care, including management of respiratory complications [6, 33]. Improving the implementation of locally appropriate technologies for targeted neonatal care, scaling up intensive care, improving respiratory complication management, newborn thermal care, breastfeeding and kangaroo mother care counselling and the diagnosis and management of neonatal jaundice and sepsis have great potential to further reduce NMR. Mapping these results to the roadmap provided by historical modelling allows us to see how the gaps found in our facility assessment maps onto key areas of improvement needed to accelerate progress.

Our study also highlighted facility capacity challenges in the low-resource health settings. These include staff and essential equipment and supply shortages, gaps in collaboration from lab technicians to nurses to clinical supervisors, lack of reliable running water and electricity and inability to diagnose infections. These must be overcome for Malawi to achieve further declines in NMR. A cluster randomized controlled trial in paediatric hospitals in Kyrgyzstan demonstrated the ability of a similar WHO tool to improve adherence to WHO guidelines and overall quality of care, which highlights the quality improvement potential of facility assessments [34].

### Using the assessment tool: limitations and lessons learned

There were a number of data collection challenges to implementing the facility assessments. One of the previous recommendations to improve the WHO integrated quality of care assessment tool was to shorten the assessment [13]. Though the hospital visit is intended to be conducted over approximately 2 days [20, 21], our data collectors reported that it was challenging to find staff for interviews, particularly the district supervisors, and we found that the assessment took up to 2 weeks to complete even reduced to only the neonatal care components. This highlights some of the challenges of low-resource health settings where availability of personnel is a significant factor. Additionally, there was some pushback from hospital staff who did not see any direct benefits of the survey. They felt that it was only time consuming, which is understandable in understaffed units. Extracting data from the registries for background statistics information on the health facility was also challenging because data was poorly entered in the register, with missing and torn pages, and there was some confusion between district and facility level data. Documentation was found to be a critical gap in understanding quality of care at the health facilities.

Additionally, there were concerns with the checklist methodology of the assessment tool; values were weighted equally and some scores were artificially inflated by questions of less value. For example, while the case management of sick newborns required some improvement to meet standards of care, the overall score hid critical low scores in individual questions around management of convulsions, diagnostics of infection and monitoring glucose levels. Additionally, while the calculated monitoring and follow-up of sick neonates score was in the high threes across the three district hospitals, a closer examination finds that this is largely driven by follow-up by nurses but hiding a critical gap in reassessment by physicians.

Furthermore, there was ambiguity in some of the topic areas due to lack of specificity of the questions. For example, it was unclear which specific components of kangaroo mother care were practiced beyond a focus on skin-to-skin contact immediately after birth. While there was one question about restrictions on the frequency or length of breastfeeding, no questions explored whether there was continued support for the mother to continue breastfeeding or her level of comfort around breastfeeding practices during the rest of her stay and post-discharge. Additionally, there is ambiguity around the use of the word “disinfection” and it is unclear whether it is deep or surface cleaning.

Lastly, the different perspectives from different respondents, from nurses to lab technicians to district health officers to the observations of the data collectors themselves, were consolidated into a calculated quantitative score. This masks some of the complexities and contradictions that are found as components are teased apart. Lab test times may reflect the response of the laboratory technician to run the test but not the whole diagnostic cycle from the ward to the lab. In the Malawian district hospitals covered in this study, it is of note that that the assessment tool does not integrate substantial delays due to power outages into the reported timing. Knowing tests availability at laboratory may fail to reveal crucial overall health system barriers such as reliable electricity, the whole diagnostic cycle from ward to lab, rate of ordering tests, etc. This is especially important as the WHO recently added an essential diagnostics list to accompany the essential medicines list. Consequently, qualitative research is recommended to accompany the tool in order to better understand the contexts of care and how the areas of care interact.

## Conclusion

The WHO integrated quality of care assessment tool is a validated tool, approved by WHO and is a good way of standardising facility assessments. Evaluation of the quality of neonatal care in rural southern Malawi highlighted the complex challenges faced in district-level health facilities. The results reveal that women and newborns are receiving care. However, the quality of care received needs improvement, particularly for sick and vulnerable newborns. The Essential Newborn Action Plan (ENAP) aims to reduce neonatal mortality to 15/100,000 live births by 2035, which has been integrated into the Malawian national health strategy (Health Sector Strategic Plan HSSP II 2017–2022) [35]. While our facility assessment was independently conducted, Malawi is part of the WHO Quality of Care Network and the Ministry of Health has prioritized improving the quality of maternal, newborn and child health care across the country. Results from our study will inform planning and advocacy strategies at the Malawian Ministry of Health, within study facilities as well as highlight areas where gaps exist in care, supplies and infrastructure during the scale-up of nurseries in district hospitals in Malawi. More broadly, results from our study contribute to the larger conversation in global health on reducing newborn deaths. While women are delivering at facilities at a higher rate than ever before, rural referral hospitals where women with complications are locally sent need to overcome barriers in infection control, reliable supply of essential medicines, capacity for diagnostics and improvements in newborn thermal care and management of respiratory complications for small and sick babies to accelerate neonatal mortality reductions.

## Supplementary information

**Additional file 1.** Areas of care assessed by the adapted WHO integrated quality of care assessment tool.

## Data Availability

See Additional file 1 for areas of care assessed by the adapted WHO integrated quality of care assessment tool. Any additional data will be available upon request to the corresponding author.
