# Supplementary material for: Assessing quality of newborn care at district facilities in Malawi
Source: BMC Health Serv Res. 2020 Mar 18;20:227. doi: 10.1186/s12913-020-5065-2 (PMC7079536; doi:10.1186/s12913-020-5065-2)
Supplement: Supplementary file 1 — Additional file 1. Areas of care assessed by the adapted WHO integrated quality of care assessment tool. [file 12913_2020_5065_MOESM1_ESM.docx]

**Additional file 1: Areas of care assessed by the adapted WHO integrated quality of care assessment tool**

| **Area of care** | **Standards assessed** |
| --- | --- |
| Infrastructure | Availability of electricity, back-up power supply, running water, soap/disinfectant, sharps disposal, fridge for drugs, oxygen source, complaints/ suggestions box |
| Laboratory | Lab testing possible and results available for blood glucose, haemoglobin, hematocrit (PCV), HIV, syphilis, blood gas analysis, blood grouping and crossmatch, bilirubin, Rhesus antibody, urine dipstick, urine microscopy, blood culture, full blood count testing |
| Labour and delivery facilities | Presence of adequate lighting, Examination light, Wall clock, Delivery pack, Blank partographs, Heating lamp for neonates, Towels for drying newborn babies, Oxygen source: oxygen cylinder, Oxygen source: oxygen concentrator, Oxygen source: central supply, Flow meters for oxygen, Equipment for the administration of oxygen via nasal prongs, Equipment for the administration of oxygen via catheters, Equipment for the administration of oxygen via masks, Self-inflating bags for respiratory support, Neonatal size bags and masks, Anaesthetic equipment, Normal thermometer, Sterile gloves, Sterile gauze, Foetal stethoscope, Stethescope, Sphygmomanometer, Infusion sets, Infusion pumps/dosimeters, IV catheters, Urinary catheter, Syringes, Needles, Suturing set, Suturing material, Weighing scale for baby, Cord cutting/cord clamping set, Episiotomy scissors, Vacuum extractor, Forceps, Caesarean packs, Vacuum aspirator, Delivery beds, Regular beds, Operating theatre beds, Resuscitation table, Incubator, Tracheal tubes, Newborn bag and mask size 1 for term babies, Newborn bag and mask size 0 for pre-term babies, Laryngoscope blades, Oropharyngeal airways, Breathing valves, Electric suction pump, Suction catheter, Suction bulb, single use, Suction bulb, sterilizable multi-use, Baby scales, Hot cots, CPAP, Phototherapy |
| Caesarean section facilities | Presence of adequate lighting, Examination light, Wall clock, Delivery pack, Blank partographs, Heating lamp for neonates, Towels for drying newborn babies, Oxygen source: oxygen cylinder, Oxygen source: oxygen concentrator, Oxygen source: central supply, Flow meters for oxygen, Equipment for the administration of oxygen via nasal prongs, Equipment for the administration of oxygen via catheters, Equipment for the administration of oxygen via masks, Self-inflating bags for respiratory support, Neonatal size bags and masks, Anaesthetic equipment, Normal thermometer, Sterile gloves, Sterile gauze, Foetal stethoscope, Stethescope, Sphygmomanometer, Infusion sets, Infusion pumps/dosimeters, IV catheters, Urinary catheter, Syringes, Needles, Suturing set, Suturing material, Weighing scale for baby, Cord cutting/cord clamping set, Episiotomy scissors, Vacuum extractor, Forceps, Caesarean packs, Vacuum aspirator, Delivery beds, Regular beds, Operating theatre beds, Resuscitation table, Incubator, Tracheal tubes, Newborn bag and mask size 1 for term babies, Newborn bag and mask size 0 for pre-term babies, Laryngoscope blades, Oropharyngeal airways, Breathing valves, Electric suction pump, Suction catheter, Suction bulb, single use, Suction bulb, sterilizable multi-use, Baby scales, Hot cots, CPAP, Phototherapy |
| Prevention and management of preterm labour | Management of preterm labour and antenatal administration of corticosteriods |
| Nursery facilities | Availability of toilets, access to clean running water, clean and safe beds, mosquito nets, ward is clean, sharps container |
| Infection control | Hand hygiene, use of glovers, infection control practices |
| Supportive care of sick neonates | Supportive care including IV fluids use, drug treatment and blood transfusion |
| Neonatal care equipment and supplies | Availability of incubator, radiant warmer, a heated mattress cot/hot cot, phototherapy lamp, appropriate sized Ambu bag, oxygen supply/concentrator, appropriate sized face mask available, CPAP system, multi-function monitor, pulse oximeter, nasogastric tubes, glucometer, suction apparatus, thermometer, digital weighing scale |
| Routine neonatal care | Neonatal resuscitation, newborn assessment and immediate care, examination/screening/prevention/treatment of vertically transmitted infectious diseases in the newborn, early and exclusive breastfeeding, monitoring of the newborn before discharge, information and counselling for mothers |
| Case management of the sick newborn | Management of pre-term babies and low birth weight babies, neonatal sepsis, recognition and treatment of jaundice, management of convulsions in neonates, feeding needs for sick neonates |
| Monitoring and follow-up of sick newborns | Monitoring of individual progress, monitoring by nurses, reassessment by physicians and follow-up |
